# Monstrosities

**Published:** 1853-04

**Authors:** 


### MONSTROSITIES.

It is now well understood that the fetuses born with defective organization, constituting the "monsters" of which so many cases are on record, are examples of *arrest of development.* The hermaphrodites of the older and later writers, come under this head. It has long been and is still a question, how far the imagination of the mother is influential in the production of such monsters. A curious case of arrest of development is given, by Dr. R. Lee Fearn, in the Proceedings of the Medical Association of the State of Alabama, for December, 1850, in which the impressions of the mother would seem to have affected the child in utero. It is as follows:

"A gentleman, whilst shooting, shot through the metacarpal bone of his index finger. The wound was a bad one, and piece after piece of the bone came away. A few months after the accident here mentioned, and in due season, his wife bore him a child perfectly formed in all respects. When about four months advanced in her second pregnancy, an operation was deemed necessary to remove the last remaining portion of bone in her husband's finger. She witnessed the operation, and was much shocked and alarmed at the sight. When her child was born, it was found to be deficient in this very bone, though in all other particulars it was a well-formed child."

